# Uptake and effectiveness of the Children's Fitness Tax Credit in Canada: the rich get richer

**DOI:** 10.1186/1471-2458-10-356

**Published:** 2010-06-21

**Authors:** John C Spence, Nicholas L Holt, Julia K Dutove, Valerie Carson

**Affiliations:** 1 Faculty of Physical Education & Recreation, University of Alberta, Edmonton, Alberta, Canada; 2 Kinesiology and Health Sciences, Queen's University, Kingston, Ontario, Canada

## Abstract

**Background:**

The Government of Canada implemented a Children's Fitness Tax Credit (CFTC) in 2007 which allows a non-refundable tax credit of up to $500 to register a child in an eligible physical activity (PA) program. The purposes of this study were to assess whether the awareness, uptake, and perceived effectiveness of this tax credit varied by household income among Canadian parents.

**Methods:**

An internet-based panel survey was conducted in March 2009 with a representative sample of 2135 Canadians. Of those, parents with children aged 2 to 18 years of age (*n *= 1004) were asked if their child was involved in organized PA programs (including dance and sports), the associated costs to register their child in these programs, awareness of the CFTC, if they had claimed the CFTC for the tax year 2007, and whether they planned to claim it in the upcoming year. Parents were also asked if they believed the CFTC has lead to their child being more involved in PA programs.

**Results:**

Among parents, 54.4% stated their child was in organized PA and 55.5% were aware of the CFTC. Parents in the lowest income quartile were significantly less aware and less likely to claim the CFTC than other income groups. Among parents who had claimed the CFTC, few (15.6%) believed it had increased their child's participation in PA programs.

**Conclusions:**

More than half of Canadian parents with children have claimed the CFTC. However, the tax credit appears to benefit the wealthier families in Canada.

## Background

The majority of children in developed countries are insufficiently physically active to achieve health benefits [1,2]. For instance, approximately 87% of Canadian children do not meet national guidelines of 90 minutes of moderate to vigorous physical activity on a daily basis.^1 ^Furthermore, children from low-income families are more likely to be physically inactive and engage in sedentary pursuits [3]. The financial costs associated with organized physical activity (PA) programs (including sport and dance) are often a barrier for participation [4-6]. Thus, a potential policy option for governments concerned about issues of childhood physical inactivity and obesity is to alleviate the cost of participation in organized PA by offering tax rebates to families [7]. However, few examples of such policies exist in the world and even less is known about the effectiveness of these programs.

The Government of Canada implemented the Children's Fitness Tax Credit (CFTC) in 2007 that allows a non-refundable tax credit of up to $500 to register a child 16 years old or younger in an eligible PA program [8]. Depending on the net taxable income of a household this would amount to, at most, a tax saving of $75 per child. To qualify, PA programs must be offered for a minimum of 8 consecutive weeks or 5 consecutive days, be supervised, and contribute to "cardio-respiratory endurance, plus one or more of: muscular strength, muscular endurance, flexibility, or balance." [8] In the case of children with disabilities, the parent may claim an extra $500 for children up to 18 years of age and include costs for equipment, assistive devices, and transportation. The CFTC was crafted based on the principles of effectiveness, simplicity, efficiency, and equity [9]. Effectiveness relates to the tax credit promoting participation in PA that has long lasting effects. Simplicity is making the process of claiming the CFTC as easy as possible for parents, organizations, and the government. The principle of efficiency requires that the tax credit be implemented and used by parents as quickly as possible. The final principle, equity, calls for the CFTC to be beneficial to parents across Canada in all economic circumstances, and to be equally beneficial for children with disabilities.

Although Canada is the only country to have a nationwide fitness tax credit program, some provinces and territories have a tax credit, and some countries are considering creating a fitness tax credit for children and/or adults. The provinces of Manitoba and the Yukon offer a similar tax credit as the CFTC, with additional amounts for children with disabilities [10,11]. The province of Nova Scotia also offers a $500 credit, but it is available for children up to 17 years old, as long as the organization is registered with Nova Scotia Health Promotion (a government ministry) [12]. Starting in 2009, the Nova Scotia fitness tax credit will also be available for adults. Similarly, a bill was introduced in April 2009 to create a fitness tax credit in the United States. This would allow up to $1000 credit on fitness facility memberships, program registration fees, or fitness equipment for adults or children [13]. Australia is also considering a child fitness tax credit. Specific details have not yet been determined, but a credit of at least $250 has been proposed, with similar criteria as the CFTC for determining who is eligible [14]. Since governments in several countries are now considering implementing some type of tax incentive to encourage PA, it is important to examine whether these policy instruments can be effective in promoting PA among children.

A basic assumption of such tax rebates is they will help alleviate economic barriers that inhibit participation in PA. From an economic point of view, it is only rational to assume that people will make the right choice when presented with financial incentives. However, according to rational choice theory [15], people make choices while considering not only incentives and benefits but also constraints such as their budget. It seems logical that families with limited financial resources will be less likely to enroll their children in organized PA programs that have associated financial costs (e.g., registration, equipment, travel), regardless of whether a tax rebate may be claimed at the end of the year. Thus, we argue that policy instruments such as the CFTC disproportionately favor those families who enjoy sufficient wealth to afford to register their children in organized PA programs and cover the associated costs of participation [16]. Similar to the knowledge gap hypothesis [17] where PA promotion campaigns are often more effective for the educated segment of a society [18], such policy instruments may actually increase the gap in PA participation between poor and wealthy children and violate expectations of equity. Unfortunately, we are unaware of any research or evaluations of tax rebates that promote PA. In fact, recent calls have been made for research on the effectiveness of incentives such as tax rebates to encourage PA [19,20]. Therefore, the purpose of this study was to assess the awareness, uptake, and perceived effectiveness of the CFTC by Canadian parents. Specifically, we hypothesized that income would be a significant moderator of whether children would be enrolled in organized PA, and whether their parents were aware of, and claimed the CFTC.

## Methods

### Participants

These results are based on an online survey of 2,135 Canadian adults (52% female; mean age = 46.34 years) recruited through the Canadian Ipsos Reid Online Omnibus with a response rate of 40%. Consistent with national data [21], median household income for the sample was $55,000-$59,000 and the majority of respondents reported having at least a post secondary education. Also, 1,004 were parents of children aged 2-18 years. Interviews were conducted between March 3^rd ^and March 6^th^, 2009. The final data are statistically weighted to reflect the actual age, gender and education of the Canadian population and are balanced by region. With a sample of 2,135 people, one can say with 95% certainty that the overall results are within ± 2.1 percentage points of what they would have been had the entire Canadian population been surveyed. Similarly, the margin of error on 1,004 interviews is ± 3.1 percentage points. The Canadian Ipsos Reid Online Omnibus is a panel survey that includes 185,000 Canadians who have already consented to participating in survey research before joining the panel. For participating in surveys, panel members have opportunities to win prizes and accumulate tickets that then can be used to purchase other prizes. All study procedures were approved by the Faculty of Physical Education and Recreation Research Ethics Board at the University of Alberta.

### Measures

Respondents completed a brief survey including demographics (age, gender, education, household income, region of residence) and 8 items about the CFTC. Specifically, respondents were asked if they had children between the ages of 2 and 18 years. If so, they were then asked if their child(ren) was involved in organized PA programs (including dance and sports), the associated costs to register their child in these programs ($0, less than $100, $100-$499, over $500), awareness of the CFTC, if they had claimed the CFTC in 2007, and whether they planned to claim it in the upcoming year. Parents were also asked if they believed the CFTC had led to their child being more involved in PA programs and whether they had ever used any other services (e.g., subsidized municipal programs) or incentives to reduce the costs of registering their child in organized PA programs. Depending on if the respondents had children, the time required to complete the survey was 10 minutes at most.

### Data Analysis

Analyses were performed using SPSS 17. Frequencies were calculated for the awareness and claiming of CFTC (2007 & 2008) for all Canadians, parents with children aged 2 to 18 years of age, and for parents with children in organized PA. Chi-square analyses were then conducted to determine associations between household income and awareness and claiming of CFTC (2007 & 2008) among parents with children between the ages of 2 to 18 years. Associations were also examined for household income and the access of services to reduce the costs of participation among parents who had enrolled their children in organized PA; and whether parents who had claimed the CFTC for 2007 believed it had increased PA participation of their child. Finally, four binary logistic regression models were then constructed to test our hypotheses in which the sport status of the child, parental awareness of the CFTC, and claiming of the CFTC (2007 & 2008) were regressed separately on household income, while controlling for sex, age, educational level of the parent, and region of residence. For all analyses an alpha of *p *< .05 was used to determine statistical significance.

## Results

Among the sample of 2135 respondents, 47% were parents with children between the ages of 2 to 18 years and 25.6% reported having children enrolled in organized PA programs that would be eligible for the CFTC. As presented in Table 1, less than half of respondents were aware of the CFTC (42.8%), had claimed it for 2007 (12.3%), or planned to claim it for the 2008 tax year (15.5%). Among parents with children between the ages of 2 to 18 years, 54.4% stated their child was enrolled in organized PA and they appeared to have higher levels of awareness of the CFTC (55.5%), claims for 2007 (26.1%), and plans to claim for 2008 (33.1%) than Canadians in general. Similarly, parents with children in organized PA programs, demonstrated high levels of awareness of the CFTC (64.9%), claims for 2007 (41.8%), and plans to claim for 2008 (52%). For parents with children between the ages of 2 to 18 years, significant unadjusted positive associations were observed for household income and whether a child was enrolled in organized PA, *χ *^*2 *^(3, 1005) = 50.11, *p *< .0001, awareness of the CFTC, *χ *^*2 *^(3, 951) = 58.48, *p *< .0001, claimed CFTC for 2007, *χ *^*2 *^(3, 557) = 26.37, *p *< .0001, and planned to claim CFTC for 2008, *χ *^*2 *^(3, 516) = 37.71, *p *< .0001. For instance, 28.2% of parents in the lowest income quartile had claimed the CFTC for the 2007 tax year while approximately 55% of parents in the highest income quartile had claimed it. Similarly, a positive association was observed between household income and the average amount of money parents reported spending to register their child in organized PA, *χ *^*2 *^(9, 1003) = 119.19, *p *< .0001 (see Table 2).

**Table 1:** Proportion (%) of Canadians with Children Involved in Organized PA and their Level of Awareness and Uptake of the Children's Fitness Tax Credit (CFTC).

	Child in Organized PA	Aware of CFTC in 2009	Claimed CFTC in 2007	Plan to Claim CFTC for 2008
Total sample (*N *= 2135)	25.6	42.8	12.3	15.5
Parents with children aged 2 to 18 years (*N *= 1004)	54.4	55.5	26.1	33.1
Parents with children in organized PA		64.9	41.8	52.0
(*N *= 546)				
Household Income^a^				
Lowest quartile	40.1	38.8	28.2	40.3
2	45.9	56.5	36.9	57.3
3	59.0	62.5	55.7	76.5
Highest quartile	67.7	71.9	54.8	69.5

PA = physical activity, sport, or dance.

^a^For parents with children aged 2 to 18 years.

**Table 2:** Proportion (%) of Canadian Parents Reporting Average Amount of Money Spent per Year to Register Children in Sport, Physical Activity, or Dance by Household Income.

	$0	Less than $100	$100 to $499	$500 or more
Lowest income quartile	39.8	23.8	27.7	8.7
2	33.6	20.5	34.1	11.8
3	20.9	14.5	35.9	28.6
Highest income quartile	12.9	11.0	45.3	30.7

After adjusting for sex, age, and education level of the parent along with region of residence in a series of logistic regressions, significant associations were observed between household income and whether a child was enrolled in organized PA, *χ *^*2 *^(13, 951) = 83.01, *p *< .0001, aware of the CFTC, *χ *^*2 *^(11, 951) = 82.13, *p *< .0001, claimed CFTC for 2007, *χ *^*2 *^(11, 557) = 31.48, *p *< .0001, and planned to claim for 2008, *χ *^*2 *^(11, 516) = 42.23, *p *< .0001 (see Table 3). For example, parents in the highest-income quartile were more likely than parents in lower income households to report their child being involved in organized PA (OR = 2.49) and that they were aware of the CFTC (OR = 4.10), had claimed it for 2007 (OR = 2.96), and were planning to claim it for 2008 (OR = 3.14).

**Table 3:** Associations between Household Income and Child Involvement in Organized Physical Activity (PA), Awareness, and Claiming of the Children's Fitness Tax Credit (CFTC) among Canadian Parents (*N *= 1004)λ.

	Child in Organized PA		Aware of CFTC in 2009		Claimed CFTC in 2007		Plant to Claim CFTC for 2008	
	OR	95% CI	OR	95% CI	OR	95% CI	OR	95% CI
Lowest income quartile	1.00		1.00		1.00		1.00	
2	1.18	0.81-1.73	2.10	***1.42-3.10	1.43	0.78-2.61	1.93	*1.05-3.56
3	1.88	**1.28-2.76	2.85	***1.92-4.25	3.04	***1.68-5.50	4.48	***2.37-8.45
Highest income quartile	2.49	***1.70-3.64	4.10	***2.75-6.13	2.96	***1.66-5.30	3.14	***1.72-5.72

Adjusted for sex, age, and education level of the parent and region of residence.

* *p *< .05

** *p *< .001

*** *p *< .0001

Of those parents who had enrolled their children in organized programs, 15.4% reported accessing services (e.g., low-cost or subsidized programs) to reduce the costs associated with participation. Families in the lowest income quartile were much more likely to report using these services (32%) than families from higher income quartiles (16.3%, 11.6%, 10%), *χ *^*2 *^(3, 545) = 26.57, *p *< .0001.

Among parents who had claimed the CFTC for 2007, 15.6% agreed the CFTC had increased their child's participation in organized PA. Level of agreement varied by household income, *χ*^*2 *^(3, 262) = 14.69, *p *= .002, with those in the lowest-income quartile (37.5%) being much more likely to agree the CFTC increased their child's PA than those in the second (24.4%), third (11.5%), or highest-income quartile (10.4%).

## Discussion

This study is the first to examine the uptake and effectiveness of a tax credit to increase PA levels of children. As hypothesized, we found household income was a significant factor in whether Canadian parents were more likely to report their child being in organized PA, and if the parent was more likely to be aware of and claim the CFTC. It appears a tax credit such as the CFTC will only benefit those people who can afford to pay the costs of registration for a PA program and carry that burden through to the end of the tax year. This observation is supported by the fact that approximately 63% of parents from low-income households reported spending $0 to less than $100 on their child's registration for PA while 76% of parents in the highest-income quartile spent more than $100 with 31% spending $500 or more. These findings are in contrast to the Government of Canada's objective that parents from "different circumstances" have equitable opportunity to benefit from the CFTC [9]. Thus, unless some other arrangement is made, this tax credit can only be inequitable for a large segment of the population.

On the question of effectiveness, approximately 16% of parents who had claimed the CFTC agreed it had increased their child's participation in organized PA. This level of agreement ranged from 37.5% among low-income families to 10.4% among the highest-income families. Thus, even though children from low-income families are less likely to be enrolled in organized PA, and their parents are less likely to have claimed the CFTC, the tax credit appears to be most effective for increasing PA among such children. Therefore, if a more equitable mechanism can be devised to allow low-income families to take advantage of the CFTC, it is possible the tax credit can be an effective policy instrument for encouraging PA among children. However, our research provides no insight on whether the children who are benefiting from the tax credit are already sufficiently active. Therefore, more research is required to determine who is benefitting the most from the CFTC.

Though the CFTC is an example of an economic intervention to promote PA [16], economics is solely concerned with efficiencies associated with policies and does not consider social values such as fairness and equity [20,22]. Therefore, if a tax credit is to be effective for all children, then alternative solutions need to be sought for dealing with issues of inequity. In making their recommendation for the CFTC to the Canadian Government, the Expert Panel for the Children's Fitness Tax Credit recognized cost of programs as a potential barrier and suggested "exploring sponsorship opportunities for people who might choose to help children whose parents do not have the means to pay for membership fees, programs, or camps on their own." [[9]; p. 25] Unfortunately, the Canadian Government did not act on this recommendation when the CFTC was initiated. Based upon our findings, the government should reconsider the recommendation of the expert panel and attempt to identify organizations that could sponsor low-income children's participation in organized PA. For example, not-for-profits and municipalities often provide financial support or no-cost programming for children in need so they can participate in various activities. Approximately one-third of low-income families with children in organized PA reported they used such programs to reduce the costs of registration. Instead of expecting these organizations to be solely responsible for raising funds to support the PA of these children, perhaps the Canadian Government should consider allocating funds to such organizations in the amount equivalent to a tax credit for each child sponsored.

To address the gap in awareness of the tax credit between low- and high-income families, one solution could be more and relevant promotion of the CFTC to low-income families. For instance, instead of relying on tax guides and government websites, materials about the tax credit could be distributed at public health centers and schools in low-income neighborhoods and regions across the country. But, until such families are able to afford the costs of registering their children in organized PA lack of knowledge and awareness of the CFTC will be a secondary issue.

This study is not without limitations that should be acknowledged. For instance, the cross-sectional nature of the study limits our ability to establish cause-and-effect relationships. Second, self-reports of behavior are susceptible to bias and it is possible the parents responding to our survey were more positively predisposed to the CFTC. This may have been compounded by the fact that our participants were recruited from a panel survey where they received some form of reimbursement for their engagement. Therefore, future research is required to determine the accuracy of the reported levels of claiming the CFTC in our study. For instance, it would be useful if the Canada Revenue Agency could provide some descriptive information (e.g., household income) on the proportion of claims made for the CFTC. Finally, due to limited funds, we did not ask about the disability status of children. Since disability is another potential source of inequity that the Canadian Government has included under their principle of equity for the CFTC, future research should examine whether the tax credit has increased opportunities for children with a disability to engage in organized PA.

## Conclusions

Household income is an important determinant of whether Canadian children engage in organized PA and whether their parents are aware of and claim a tax credit to subsidize this participation. Basically, families at the lower end of the income continuum cannot afford the costs associated with organized PA and are less likely to be able to take advantage of a tax credit. If other countries and jurisdictions wish to implement a similar tax credit to encourage PA, then some consideration should be given to preventing the potential inequities that may arise. Though children may not be able to make rational choices about participation in PA [16], parents and governments do have influence over the environments in which these opportunities may exist [23]. Therefore, it is important that programs and policies that are implemented with the intent to encourage PA of children do not favor those who are already in an advantaged situation.

## Competing interests

The authors declare they have no competing interests.

## Authors' contributions

All authors were involved in various stages of study design. JCS and NLH developed the proposal for the project. JD and VC oversaw data collection. JCS conducted the statistical analysis and wrote the paper. All authors commented on drafts and approved the final text.

## Pre-publication history

The pre-publication history for this paper can be accessed here:

http://www.biomedcentral.com/1471-2458/10/356/prepub
